# Single-incision laparoscopic partial splenectomy for benign pediatric splenic tumors: a retrospective comparative study

**DOI:** 10.3389/fped.2025.1633940

**Published:** 2025-07-22

**Authors:** Ran Tang, De-Cheng Wei, Cheng-Xiao Zhou, Zhong-Ce Li, Jian Bian, Shi-Qin Qi

**Affiliations:** Department of Pediatric Surgery, Anhui Provincial Children’s Hospital, Hefei, China

**Keywords:** partial splenectomy, children, benign splenic tumors, single-incision, thrombocytosis

## Abstract

**Background:**

The indications for laparoscopic partial splenectomy (LPS) in pediatric benign splenic tumors are well established, but concerns remain regarding its technical complexity and potential complications. This study aimed to evaluate the safety and outcomes of single-incision LPS (SILPS).

**Methods:**

A retrospective analysis was conducted on 22 children who underwent SILPS from July 2021 to April 2024, compared with 25 patients who received laparoscopic total splenectomy (TS). Clinical characteristics, operative details, and postoperative outcomes were assessed.

**Results:**

SILPS patients had comparable operative time, blood loss, and hospital stay to those in the TS group. However, SILPS was associated with significantly lower rates of postoperative thrombocytosis and leukocytosis. No major perioperative complications were observed.

**Conclusion:**

SILPS is a safe and effective spleen-preserving technique for pediatric benign splenic tumors, offering reduced hematologic complications without increasing surgical risks. It is technically demanding and requires experienced laparoscopic skills and proper patient selection.

## 1 Introduction

Benign tumors of the spleen, including splenic hemangiomas, splenic lymphangiomas, and splenic cysts, often present with no significant symptoms in the early stages. However, as the condition advances, it can impair spleen function and result in compression of surrounding organs, leading to associated symptoms. Therefore, timely diagnosis and proper treatment are imperative (1). Laparoscopic splenectomy is considered the most effective treatment for splenic tumors (2). However, recent studies have highlighted the spleen's significance as the largest lymphoid organ in the body, constituting about 25% of total lymphoid tissue (3). It serves as a vital immune organ, housing numerous lymphocytes and macrophages (4). Research has confirmed its pivotal role in infection resistance, immune regulation, anti-tumor activity, and hematopoiesis (5, 6). The importance of preserving spleen function, particularly for its anti-infection and anti-tumor activities, has been increasingly recognized. Complete splenectomy may predispose patients to infections. While partial splenic artery embolization addresses complications post total splenectomy, it can also lead to abscesses, splenic rupture, and post-embolization complications. Therefore, partial splenectomy is a favorable option for specific patients, with spleen function preservation significantly reducing complications associated with total splenectomy (7, 8).

Partial splenectomy (PS) was first described in the early 20th century, but its widespread adoption was limited by technical challenges and the risk of postoperative hemorrhage. With advances in surgical techniques and intraoperative hemostasis, PS gained renewed interest in the 1990s (2), particularly in pediatric patients, where preservation of splenic immune function is critically important. Today, PS is increasingly employed in the management of benign splenic lesions and certain hematologic conditions, especially when tumors are localized to the poles of the spleen and splenic preservation is feasible.

Laparoscopic partial splenectomy (LPS) is increasingly utilized for benign splenic tumors and traumatic splenic ruptures. With rising patient demand for aesthetically pleasing surgical outcomes, single-incision laparoscopy is now being explored for partial splenectomy in pediatric patients with splenic benign tumors.

## 2 Materials and methods

### 2.1 General information

This retrospective cohort study supported by the The Medical Research Ethics Committee of Anhui Provincial Children's Hospital (No. EYLL-2024-005) and conformed to the ethical guidelines of the 1975 Declaration of Helsinki. The work has been reported in line with the STROCSS criteria (9). We conducted single-incision laparoscopic partial splenectomy on 22 (PS) patients with various splenic lesions, comparing them with 25 patients who underwent laparoscopic total splenectomy (TS) by the same surgical team during the same period. Our aim was to evaluate the safety and clinical efficacy of single-incision laparoscopic partial splenectomy in treating benign splenic tumors. This study collected data from 22 patients with benign splenic tumors treated at Anhui Children's Hospital between July 2021 and March 2024, including 10 cases of splenic hemangioma, 5 cases of splenic lymphangioma, and 7 cases of splenic cysts. All patients underwent single-port laparoscopic partial splenectomy (PS). Inclusion criteria for the PS group were: (1) Preoperative CT or ultrasound diagnosis of a benign splenic tumor; (2) Tumor diameter >3 cm; (3) Tumor located in the upper or lower pole of the spleen. Exclusion criteria were: (1) Suspected malignant splenic tumor; (2) Multiple lesions in the spleen; (3) Tumor located near the splenic hilum; (4) Severe comorbidities of the heart, lungs, or other major organs; (5) Abnormal coagulation function. Informed consent was obtained from the guardians of all included cases. Additionally, 25 patients who underwent single-port laparoscopic total splenectomy (TS) performed by the same surgical team during the same period were selected as the comparison group. It should be noted that this was not a strictly controlled trial, as the inclusion criteria for the TS group were not identical to those of the PS group. Instead, the TS group was used as a clinical reference group to reflect real-world practice. Inclusion criteria were: (1) preoperative CT or ultrasound diagnosis of a mid-splenic tumor or a tumor near the splenic hilum; (2) hereditary spherocytosis meeting surgical criteria; (3) thalassemia. Among them were 6 cases of benign splenic tumors, 3 cases of thalassemia, and 13 cases of hereditary spherocytosis. Exclusion criteria were: (1) massive splenomegaly (diameter >12 cm); (2) abnormal coagulation function; (3) severe comorbidities of the heart, lungs, and other major organs.

### 2.2 Surgical procedure

**Figure 1:**
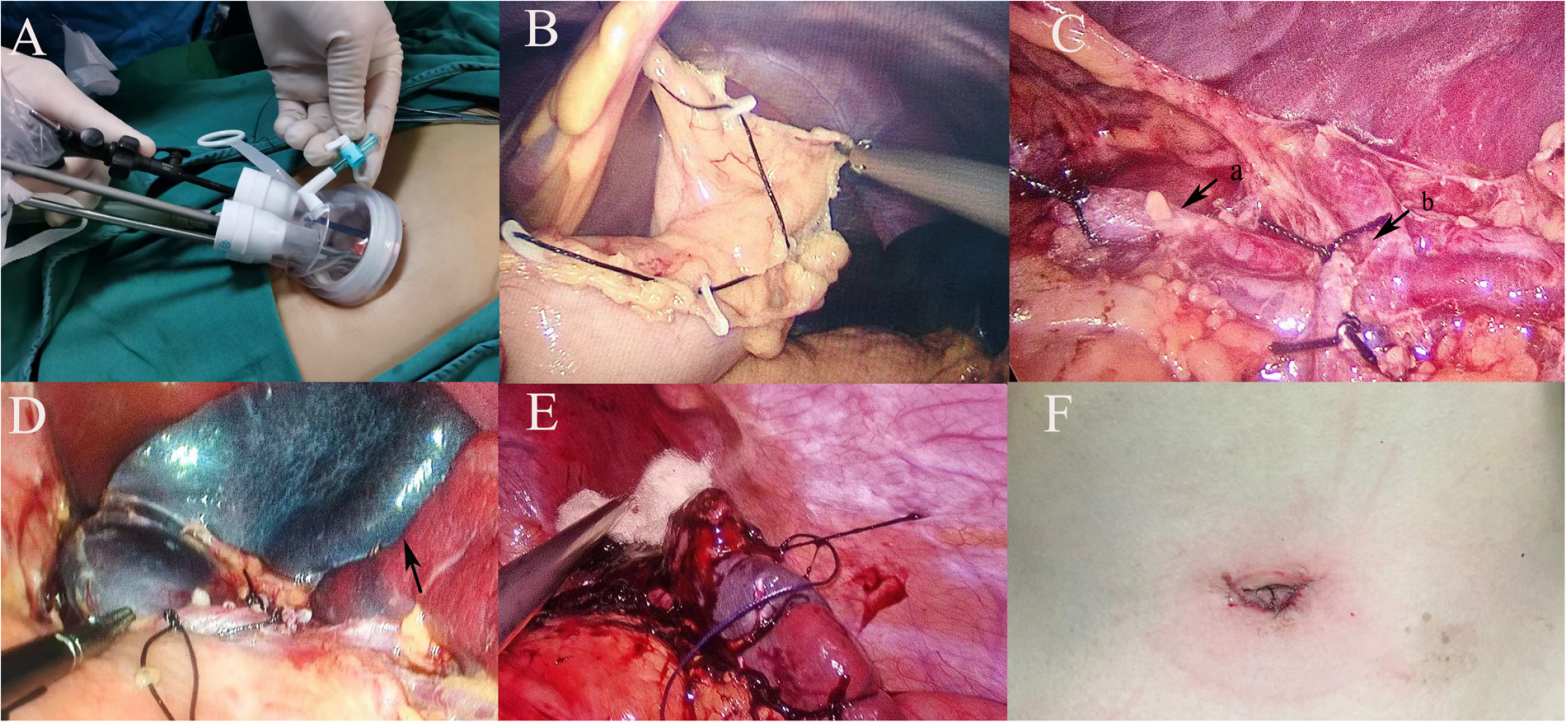
**(A)** Make a 2–3 cm incision below the navel and perform the procedure using a single-port puncture instrument; **(B)** suspend the gastric ligaments around the stomach; **(C)** dissect the splenic hilum, (a) suspend the splenic artery trunk with a thread; (b) ligate secondary vessels according to the tumor's location; **(D)** wait for the splenic ischemic line to form after ligating the vessels (black arrow); **(E)** fix the remaining spleen to the abdominal wall after hemostasis using bipolar electrocoagulation and hemostatic materials; **(F)** postoperative incision appearance.

After the induction of general anesthesia, the patient was placed in a supine position with the left subcostal area elevated. The surgeon stood between the patient's legs, the laparoscope was positioned on the patient's left side, and the assistant stood on the patient's right side. A 2–3 cm arc-shaped incision was made at the lower margin of the umbilicus, and a single-port trocar (Schneider, Figure 1A) was used to access the abdominal cavity. Carbon dioxide pneumoperitoneum was established, maintaining pressure at 10–12 mmHg. After entering the abdominal cavity, the liver, gallbladder, pancreas, stomach, and intestines were inspected. The splenic ligaments were then mobilized, and the location of the splenic tumor was identified via laparoscopy. The gastric wall or perisplenic ligaments were suspended using 2-0 silk sutures (Figure 1B) to fully expose the spleen, and, if necessary, the spleen itself was suspended. The splenic artery was identified along the upper edge of the pancreas, and the surrounding tissue at the splenic hilum was dissected using an ultrasonic scalpel to expose the secondary branches of the splenic artery. The main trunk of the splenic artery was isolated and suspended with a silk suture (Figure 1C). Depending on the tumor location, the upper or lower polar branches of the splenic artery were precisely ligated (Figure 1C), and the ischemic demarcation line was awaited to confirm the tumor was within the ischemic region (Figure 1D). The ultrasonic scalpel was used to cut 0.5–1.0 cm from the ischemic line toward the lesion, performing the partial splenectomy. Bipolar electrocautery was then used for hemostasis on the remaining splenic tissue. Biological hemostatic material was applied to the surface of the splenic remnant. The remaining spleen was optionally fixed to the abdominal wall (Figure 1E). The surgical specimen was removed through the umbilical incision, and the operation was concluded after confirming that there was no active bleeding in the splenic remnant.

For cases with significant intraoperative bleeding or difficulties with single-port laparoscopic surgery, an additional 5 mm trocar was inserted in the left upper abdomen (single port plus one), allowing the assistant to help improve exposure of the surgical field.

### 2.3 Statistical analysis

The whole cohort was divided in two groups according to the type of surgical approach used (PS vs. TS). Continuous variables were presented as median and interquartile range (IQR) and were compared using Mann-Whitney *U* test. Categorical variables were expressed as numbers and percentages and were compared with Fisher Exact test. A *p*-value of <0.05 was considered significant. Statistical analysis was performed with PRISM 8.2.0 (GraphPad Software Inc. USA) and SPSS software 23.0 (SPSS, Inc. USA).

## 3 Results

### 3.1 Patient database

From July 2021 to August 2024, a total of 22 children with benign splenic tumors underwent laparoscopic partial splenectomy (PS) at Anhui Provincial Children's Hospital. During the same period, 25 children underwent laparoscopic total splenectomy (TS). Among the PS group, 3 cases (13.6%) were completed using the single-port plus one technique, while the remaining 20 cases were completed with single-port laparoscopy, with no cases requiring conversion to open surgery. Postoperative pathological diagnoses included 10 cases of splenic hemangioma, 5 cases of splenic lymphangioma, and 7 cases of splenic cysts, with an average age of 9.36 ± 2.77 years. In the TS group, among the 25 cases, there were 6 cases of benign splenic tumors (3 splenic cysts, 1 splenic lymphangioma, and 2 splenic hemangiomas), 3 cases of thalassemia, and 16 cases of hereditary spherocytosis, with an average age of 10.2 ± 2.0 years. Two cases (8.0%) in the TS group were completed using the single-port plus one technique, while the rest were completed with single-port laparoscopy (Table 1).

**Table 1:** Patient characteristics.

Variables	Partial splenectomy (PS)	Total splenectomy (TS)	*P*-value
	*N* = 22	*N* = 25	
Gender			
Female	10 (45.4%)	13 (52.0%)	0.876
Age(year)	9.18 ± 1.85	10.2 ± 2.0	0.084
Hospitalization days	6.82 ± 0.78	7.28 ± 0.83	0.061
Main diagnosis			
Splenic cyst	7	3	
Splenic lymphangioma	5	1	
Splenic hemangioma	10	2	
Hereditary spherocytosis		13	
Thalassemia		3	

### 3.2 Comparison of operation time, intraoperative blood loss, and postoperative hospital stay

In the PS group, the average operative time was 186.77 ± 40.99 min, and the average intraoperative blood loss was 34.41 ± 6.12 ml. In the TS group, the average operative time was 160.60 ± 51.31 min, and the average intraoperative blood loss was 32.72 ± 5.20 ml. Comparisons between the two groups showed no significant differences in operative time (*p* = 0.068) or intraoperative blood loss (*p* = 0.322) (Table 2). There was no significant difference in postoperative hospital stay between the two groups (*p* = 0.018) (Table 1).

**Table 2:** Summary of postoperative outcomes.

Variables	Partial splenectomy (PS)	Total splenectomy (TS)	*P*-value
	*N* = 22	*N* = 25	
Operative time(min)	186.77 ± 40.99	160.60 ± 51.31	0.068
Intraoperative blood loss(ml)	34.41 ± 6.12	32.72 ± 5.20	0.322
Postoperative complications			
OPSI	0	1	1.0
Portal vein thrombosis	1	5	0.252
Pancreatic leak	0	0	NA
Sepsis	0	0	NA
Pulmonary infection	2	3	1.0
Subphrenic abscess	2	3	1.0
Thrombocytosis	4	19	<0.01
Conversion to single port plus one surgery	3 (13.6%)	2 (8%)	1.0

The OPSI, pulmonary infection, and subphrenic abscess variables were analyzed using Fisher's exact test, while thrombocytosis, portal vein thrombosis was analyzed using the Chi-square test. N/A, not applicable.

### 3.3 Comparison of postoperative complications

All patients completed follow-up, ranging from 12 to 20 months. In the PS group, no tumor recurrence was observed, and all patients recovered smoothly postoperatively without any cases of splenic infarction, intra-abdominal hemorrhage, sepsis, or mortality. In the TS group, 5 cases of portal vein thrombosis were reported, while 1 case occurred in the PS group, with no statistically significant difference between the two groups (*p* = 0.252). One case of overwhelming post-splenectomy infection (OPSI) occurred in the TS group. There was no significant difference in the incidence of postoperative pulmonary infections or subphrenic abscesses between the two groups (Table 2).

**Figure 2:**
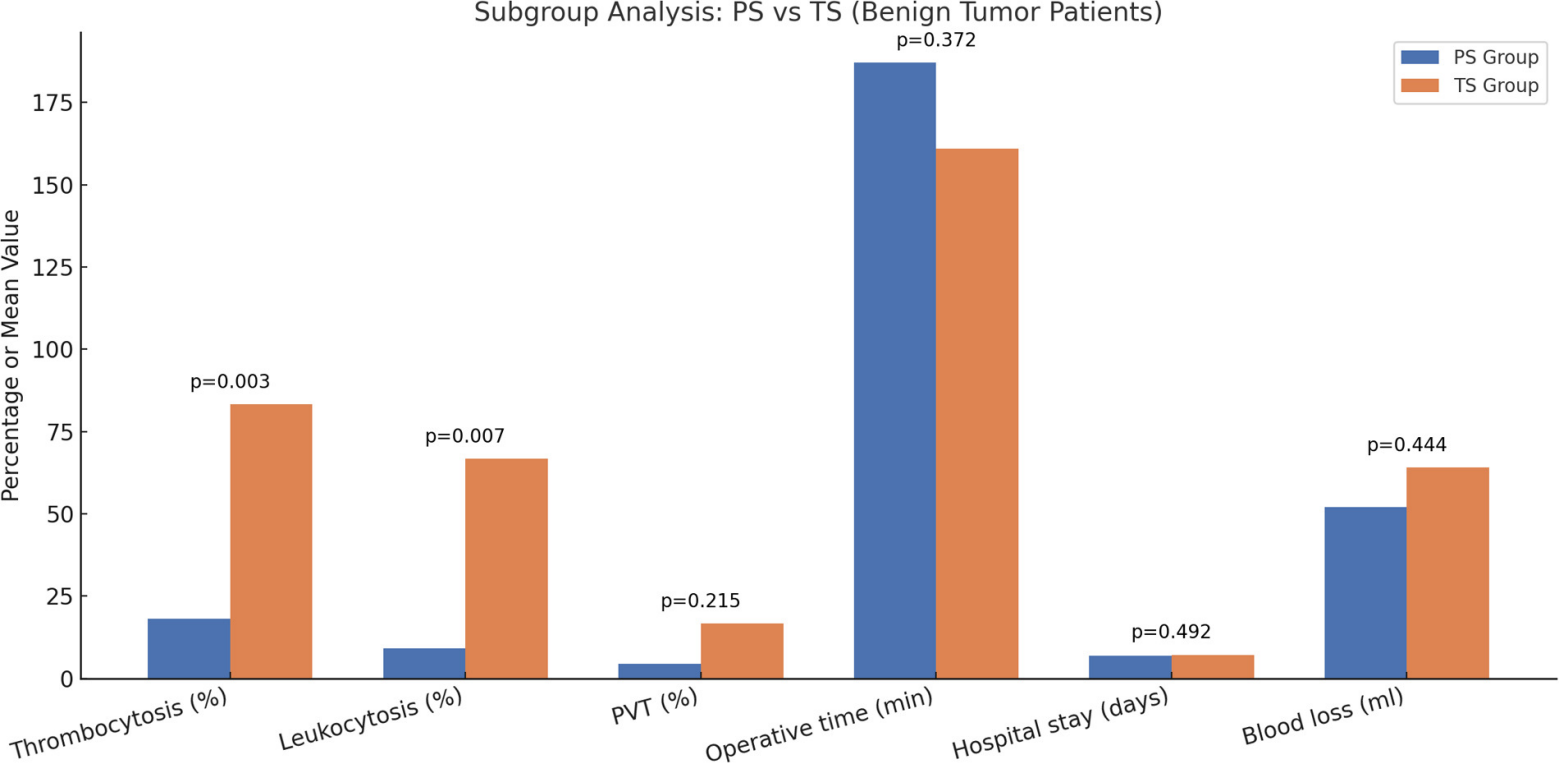
Subgroup analysis comparing postoperative outcomes between PS and TS groups in children with benign splenic tumors. The vertical axis (Value) represents either the percentage of patients with complications (thrombocytosis, leukocytosis, and PVT) or the group mean for continuous variables (operative time, hospital stay, and blood loss). *P*-values are shown above each pair of bars and were calculated to assess statistical differences between the two groups. The TS group showed significantly higher rates of thrombocytosis and leukocytosis compared to the PS group. No statistically significant differences were observed between the PS and TS groups regarding the incidence of PVT, operative time, length of hospital stay, or intraoperative blood loss.

To further assess the impact of splenic disease heterogeneity between the PS and TS groups, we conducted a subgroup analysis limited to patients with benign splenic tumors in both cohorts (*n* = 22 in PS group, *n* = 6 in TS group). The results of this analysis remained consistent with the primary findings: the TS subgroup showed significantly higher rates of postoperative thrombocytosis and leukocytosis compared to the PS subgroup (*p* < 0.05). No significant differences were observed in the incidence of PVT, operative time, blood loss, or hospital stay. This analysis reinforces the potential hematologic benefits of spleen-preserving surgery while controlling for disease background (Figure 2).

**Figure 3:**
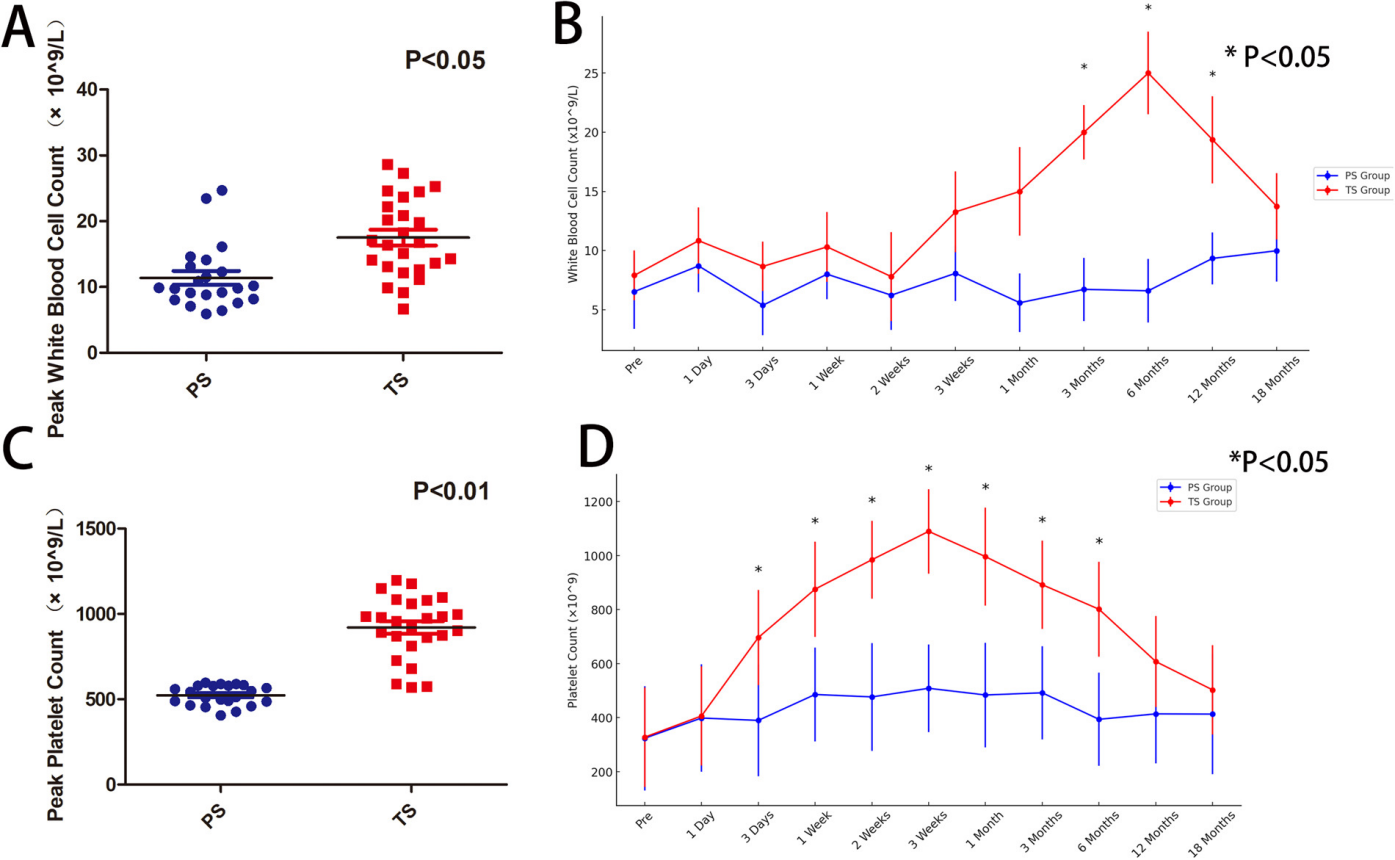
**(A)** The peak white blood cell count within 18 months postoperatively is significantly higher in the TS group compared to the PS group (*p* < 0.05); **(B)** comparison of white blood cell counts between the TS and PS groups shows that from 3 to 12 months postoperatively, the TS group had significantly higher white blood cell counts than the PS group (* indicates *p* < 0.05); **(C)** the peak platelet count within 18 months postoperatively is significantly higher in the TS group compared to the PS group (*p* < 0.05); **(D)** comparison of platelet counts between the TS and PS groups shows that from 3 days to 6 months postoperatively, the TS group had significantly higher platelet counts than the PS group (* indicates *p* < 0.05).

We analyzed the changes in platelet and white blood cell counts in both groups within 18 months postoperatively. A comparison of the peak white blood cell and platelet counts within 18 months postoperatively between the two groups showed significantly higher levels in the TS group compared to the PS group (*P* < 0.05) (Figures 3A,B). In the TS group, 19 cases of thrombocytosis (10) were observed, beginning on the third postoperative day and continuing until 6 months postoperatively, with significantly higher platelet levels in the TS group compared to the PS group. The difference disappeared at 12 months postoperatively as platelet levels in the TS group declined (Figure 3). None of the patients in either group developed postoperative OPSI. However, in the TS group, abnormal leukocytosis was noted between 3 and 12 months postoperatively, although these patients did not exhibit signs of infection or fever. The cause of abnormal leukocytosis following total splenectomy and its potential long-term effects require further investigation.

Our study indicates that partial splenectomy, compared to total splenectomy, significantly reduces postoperative increases in platelet and white blood cell counts. However, there were no significant differences between the two groups in terms of other complications such as portal vein thrombosis, pulmonary infection, or subphrenic abscess (Table 2).

## 4 Discussion

The spleen plays a crucial role in regulating blood volume, filtering blood, producing various immunoglobulins, and regulating the endocrine system (11). Patients undergoing total splenectomy are prone to several complications, such as thrombocytosis, portal phlebitis, intra-abdominal abscesses, OPSI, thromboembolism, and pulmonary hypertension (12). This is particularly concerning for pediatric patients, as early removal of the spleen can significantly reduce immunity and increase the risk of infections. Among children under the age of 5, the incidence of OPSI is 20%, which is significantly higher than in adults, and the risk of systemic infection is 60–100 times greater than in children who have not undergone splenectomy (13). Additionally, there is a lifelong risk of fatal infections after total splenectomy. Research has indicated that the incidence of portal vein thrombosis post-splenectomy can reach 50%–80%, and if not detected and treated promptly, it can lead to severe consequences (13). It is noteworthy that patients who have undergone splenectomy are in a hypercoagulable state (14), which increases the risk of thromboembolic complications not only immediately after surgery but also during long-term follow-up (15, 16). With an increased understanding of the spleen's functions, many scholars advocate preserving as much of the spleen's normal function as possible while treating splenic diseases (17). Consequently, laparoscopic partial splenectomy has gradually become a preferred method for treating certain benign splenic diseases. Furthermore, due to the advancements in minimally invasive surgery and the growing preference of patients for less invasive procedures, we have started to explore the use of single-incision laparoscopic surgery for the treatment of benign splenic diseases.

The role of partial splenectomy in treating pediatric hematologic diseases and benign splenic tumors remains controversial (18). This is due to the potential for significant perioperative complications, such as bleeding, and the possibility of splenic regrowth (19). Additionally, anatomical challenges, such as an enlarged spleen or massive splenic cysts, can make laparoscopic partial splenectomy a particularly difficult procedure (20). Due to the insufficient research on the regeneration of splenic function following partial splenectomy, we selected cases involving benign splenic tumors for this study. For hematologic diseases such as hereditary spherocytosis (HS) and thalassemia, we opted for total splenectomy. We also excluded cases of traumatic splenic rupture from our study, as the constraints of single-incision laparoscopic surgery pose higher risks for these patients. Cases with larger tumors were similarly excluded to maintain at least 25% of the normal splenic weight necessary for adequate immune function and sufficient arterial perfusion (21, 22). Thus, for cases with larger tumor diameters, total splenectomy is considered a safer option to prevent the residual spleen from being too small to maintain normal function (23).

The use of laparoscopic magnification and meticulous dissection, along with the handling of secondary splenic pedicle vessels, are crucial steps to ensure the success of the surgery. The greatest challenges in laparoscopic partial splenectomy are the anatomy of the splenic hilum, the transection of splenic parenchyma, and the management of bleeding from the splenic wound. Proper handling and appropriate vascular separation and ligation can effectively reduce intraoperative and postoperative bleeding, thereby increasing the success rate of the surgery (24). In the event of intraoperative bleeding, blind attempts to stop the bleeding should be avoided. Precise ligation of secondary vessels is the most critical step in preventing bleeding. A silk suture can be pre-looped around the main trunk of the splenic artery (24). If bleeding occurs during the handling of secondary vessels, the suture on the main trunk can be lifted to perform a temporary occlusion, allowing for rapid hemostasis. Adequate exposure of the surgical field is key to the success of the procedure. In single-incision laparoscopic surgery, due to the limitations of the single port, our experience has been to use suspension sutures to replace the assistant's help. This involves suspending the gastric wall or surrounding gastric ligaments to expose the spleen. If necessary, additional suspension sutures can be used to suspend the ligaments around the spleen or directly suspend the spleen itself to improve the surgical view. If intraoperative bleeding occurs, it is important not to attempt hemostasis blindly. Accurate vascular ligation is the most critical step in preventing bleeding. If there is significant bleeding from the splenic pedicle that is difficult to control, we recommend adding a trocar rather than insisting on a single-incision approach, which could lead to more serious consequences. For the bleeding surface of the residual spleen, some experts suggest using the “boiled meat slice” hemostasis technique to stop bleeding from the residual splenic wound (23). In our study, we found that in pediatric patients with normal coagulation function, there was minimal bleeding from the residual splenic surface after precise vascular ligation. Bipolar electrocoagulation combined with hemostatic materials was effective in stopping active bleeding within a short period.

It is important to acknowledge the heterogeneity in baseline diseases between the PS and TS groups. While the PS group exclusively included patients with benign splenic tumors, the TS group included patients with hereditary spherocytosis and thalassemia, conditions that may inherently predispose to altered hematologic parameters and thrombotic risks. Although subgroup analysis limited to tumor patients supports the primary findings, the influence of underlying disease characteristics cannot be completely excluded. Our study found that single-incision laparoscopic partial splenectomy is a safe and effective treatment for benign splenic tumors. Compared to total splenectomy, it ensures complete tumor removal while avoiding serious complications associated with total splenectomy, such as thrombocytosis and portal vein thrombosis. There were no differences between the two groups in terms of operation time, intraoperative blood loss, or postoperative hospital stay. Additionally, the single-incision approach offers aesthetically pleasing incisions, aligning with contemporary minimally invasive surgical principles. However, single-incision laparoscopic surgery is technically challenging and carries a higher risk of bleeding. The following considerations are crucial: (1) Experienced Surgeons: The procedure must be performed by experienced minimally invasive surgeons, with strict case selection. (2) Creating Surgical Space: Due to the limitation of the single port, creating adequate surgical space through repeated suspension sutures is recommended. (3) Precise Dissection and Ligation: Accurate dissection and ligation of the splenic pedicle are key to the surgery's success. If significant bleeding occurs and is difficult to control, a trocar should be added promptly to mitigate risk. (4) Identifying the Transection Line: After ligating the secondary splenic vessels, the splenic ischemia line helps determine the transection line. The cutting line should proceed 0.5–1 cm towards the lesion side from the ischemia line. (5) Preserving Sufficient Splenic Tissue: To ensure the postoperative function of the residual spleen, at least 25% of the splenic tissue should be preserved. (6) Effective Hemostasis: In children with normal coagulation function, active bleeding from the splenic surface can be quickly controlled with bipolar electrocoagulation and hemostatic materials. Repeated high-power electrocoagulation is not recommended, as its impact on the function of the remaining spleen has not been studied. These measures help enhance the safety and effectiveness of single-incision laparoscopic partial splenectomy for benign splenic tumors.

The relatively high incidence of portal vein thrombosis (PVT) in the TS group (5 cases) raised concerns regarding the safety profile of total splenectomy. However, this may be attributed to the underlying hematologic conditions—particularly hereditary spherocytosis—known to be associated with postoperative hypercoagulability. In contrast, spleen-preserving surgery like SILPS may reduce such risks by retaining partial splenic immune and hematologic regulatory functions. Nevertheless, larger-scale studies are needed to confirm this potential benefit.

In our study, detailed information regarding tumor size and location was not included. This omission was primarily due to the retrospective nature of the study, as complete imaging or intraoperative records were not available for all patients, making it difficult to accurately assess tumor dimensions and anatomical location. To avoid introducing selection bias resulting from incomplete data, we did not include this variable in the comparative analysis.

Moreover, since our study mainly focused on evaluating the safety of laparoscopic partial splenectomy (PS) and its feasibility in preserving partial splenic function compared to total splenectomy (TS), many patients in the TS group did not have splenic tumors. Therefore, we believe that a detailed analysis of tumor volume may not be necessary in the context of this study, and thus it was not performed.

## 5 Conclusion

In our study, we found that compared to patients undergoing total splenectomy, the incidence of postoperative thrombocytosis and leukocytosis was significantly lower in patients who underwent single-port laparoscopic partial splenectomy. This suggests that partial splenectomy can preserve essential splenic function without increasing the risk of other complications. An increasing body of literature and advancements in hemostatic devices indicate that partial splenectomy is the preferred treatment for patients with benign splenic tumors, as it allows for the resection of the diseased splenic tissue while preserving healthy splenic tissue and minimizing collateral damage. Furthermore, single-port laparoscopic techniques ensure effective treatment while maximizing the aesthetic outcome of the incision, aligning with current minimally invasive surgical standards.

Given the relatively small sample size and single-center design, the generalizability of our findings may be limited. Additionally, all surgeries were performed by the same skilled surgical team, raising questions about the generalizability of the technique. Nevertheless, minimizing surgical trauma while curing disease has always been a surgeon's ultimate pursuit. Future multicenter prospective studies with larger cohorts are warranted to validate these results. With the maturation of laparoscopic techniques and the advancement of laparoscopic instruments, our study confirms that laparoscopic partial splenectomy is minimally invasive, preserves the spleen's physiological function, and is safe and feasible. For early-stage adoption, it is advisable to initially perform multi-port procedures and, with increasing proficiency, transition to single-port laparoscopic surgery by selecting appropriate cases and mastering the necessary laparoscopic skills. The follow-up duration in our study (12–20 months) is relatively short and may not fully capture long-term outcomes such as splenic regrowth, functional immune status, or late-onset complications. Extended follow-up is needed in future investigations.

This study has several limitations. First, the sample size is relatively small and derived from a single institution, limiting the generalizability of the findings. Second, all procedures were performed by a highly experienced surgical team, which may not reflect the average technical capability in general practice. Third, the follow-up duration (12–20 months) may be insufficient to capture long-term complications such as splenic regrowth or late-onset thrombosis. These limitations highlight the need for future multicenter, prospective trials with longer follow-up to validate our observations.

In conclusion, single-incision laparoscopic partial splenectomy (SILPS) appears to be a safe and effective technique for the management of benign pediatric splenic tumors, with reduced hematologic complications and comparable operative outcomes to total splenectomy. Subgroup analysis supports its advantage even when controlling for disease type. However, due to the retrospective nature, small sample size, and inclusion of patients with different baseline conditions, caution is warranted in generalizing these findings. Future large-scale, prospective, and multicenter studies are essential to confirm the long-term efficacy and safety of SILPS in broader pediatric populations.

## Data Availability

The raw data supporting the conclusions of this article will be made available by the authors, without undue reservation.
